# Association between CKD-MBD and hip-bone microstructures in dialysis patients

**DOI:** 10.1093/ckj/sfae240

**Published:** 2024-08-12

**Authors:** Ken Iseri, Masahide Mizobuchi, Kanji Shishido, Noriko Hida

**Affiliations:** Department of Clinical Pharmacy, Division of Clinical Research and Development, School of Pharmacy, Showa University, Tokyo, Japan; Jinsei-kai Kasai dialysis Clinic, Tokyo, Japan; Division of Nephrology, Department of Medicine, Showa University School of Medicine, Tokyo, Japan; Internal Medicine, Sekishin-kai Kawasaki Clinic, Kawasaki, Japan; Department of Clinical Pharmacy, Division of Clinical Research and Development, School of Pharmacy, Showa University, Tokyo, Japan

**Keywords:** cortical bone, dialysis, PTH, trabecular bone

## Abstract

**Background:**

The longitudinal changes in hip-bone microstructures and estimated bone strength in dialysis patients, and the impact of chronic kidney disease–mineral and bone disorder (CKD-MBD) biomarkers on these changes, remain insufficiently explored.

**Methods:**

This retrospective study examined changes in cortical and trabecular bone compartments and estimated bone-strength indices, obtained by using 3D-SHAPER software, in the hip regions of 276 dialysis patients over up to 2.5 years. We used multivariate mixed models to investigate the associations between time-dependent CKD-MBD biomarkers and bone health metrics.

**Results:**

There was a significant decrease in areal bone mineral density (aBMD), integral volumetric BMD (vBMD), trabecular vBMD, cortical thickness and cortical surface BMD (sBMD). Similar deteriorations were found in estimated bone-strength indices [cross-sectional area (CSA), cross-sectional moment of inertia (CSMI), section modulus (SM) and buckling ratio]. Neither serum calcium nor phosphate levels were significantly associated with changes in three-dimensional parameters or estimated bone-strength indices. In contrast, serum alkaline phosphatase levels showed a significant inverse correlation with aBMD and CSA. The intact-parathyroid hormone (i-PTH) was significantly inversely correlated with aBMD, integral vBMD, trabecular vBMD, cortical thickness, cortical vBMD, CSA, CSMI and SM. When applying the KDIGO criteria as a sensitivity analysis, the higher PTH group had significant negative associations with aBMD, integral vBMD, cortical vBMD, cortical thickness and cortical sBMD. Notably, the lower PTH group showed a positive significant correlation with integral vBMD and trabecular vBMD.

**Conclusions:**

Elevated PTH, not low PTH, was associated with deterioration of hip-bone microstructures. Better management of PTH levels may play a crucial role in the hip-bone microstructure in dialysis patients.

## Graphical Abstract

**Graphical Abstract:**
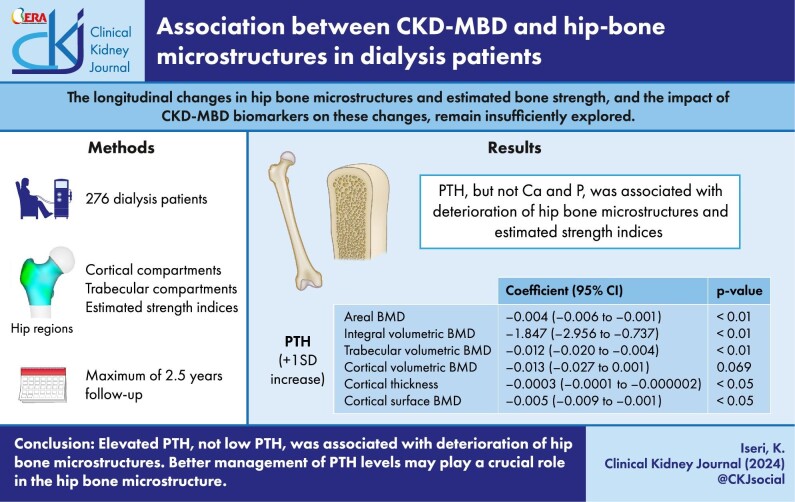

KEY LEARNING POINTS **What was known:**

- Hip fractures are the most prevalent type of fractures in dialysis patients.
- Long-term changes in the microstructures and estimated strength of hip bones, as well as the impact of biomarkers associated with chronic kidney disease–mineral and bone disorder (on these changes, remain insufficiently explored.

**This study adds:**

- Neither serum calcium nor phosphate levels showed a significant association with these changes; however, intact-parathyroid hormone (i-PTH) levels did.
- Interestingly, higher PTH group demonstrated significant negative correlations with hip-bone microstructures, while the group with lower PTH levels exhibited positive significant correlations relative to the KDIGO-recommended reference range.

**Potential impact:**

- Effective management of PTH levels may play a key role in hip-bone microstructure in dialysis patients, potentially reducing the risk of hip fractures among dialysis patients.

## INTRODUCTION

Chronic kidney disease–mineral and bone disorders (CKD-MBD), marked by disturbances in mineral metabolism including phosphate, calcium and parathyroid hormone (PTH) levels, are prevalent complications among dialysis patients. Concurrently, bone disease is also classified under CKD-MBD [1–3]. Bone disease in CKD patients exhibits a unique pathophysiology and characteristics, distinct from primary osteoporosis, which is often linked to aging and hormonal changes. In the initial stages of primary osteoporosis, trabecular bone loss occurs more rapidly than cortical bone loss [4]. Conversely, in CKD patients, a notable feature is the swift decline of cortical bone, without a similar reduction in trabecular bone [5]. However, data on the trajectory of bone health over time in dialysis patients are limited. Furthermore, while the role of intact PTH (i-PTH) in CKD-MBD management is well-established [6, 7], its impact on bone microarchitecture, particularly in a dialysis setting, has not been sufficiently explored.

Dual-energy X-ray absorptiometry (DXA) is widely used and measures the areal bone mineral density (aBMD) as a cornerstone in assessing fracture risk and diagnosing osteoporosis. Interestingly, recent advancements have enabled the development of methods for conducting three-dimensional (3D) analyses of bone via hip DXA scans [8]. In essence, these techniques, referred to as 3D-DXA, employ a 3D statistical shape and density model of the proximal femur, built from a database of quantitative computed tomography (QCT) scans. The statistical model is aligned with the DXA scan to create a patient-specific 3D model of the proximal femur, capturing its three-dimensional geometry and bone density distribution. The 3D-DXA methods have been validated against quantitative computed tomography for measuring both cortical and trabecular components, as well as estimating bone strength [9, 10]. Furthermore, 3D-DXA has been employed to assess the effects of anti-osteoporosis medication on bone disease in dialysis patients and the general population [11–13].

This study aimed to investigate the time courses of cortical and trabecular bone, as well as estimated bone strength in dialysis patients under current clinical management practices, by employing longitudinal 3D-DXA data. Furthermore, we explored the relationships between CKD-MBD parameters, particularly serum i-PTH levels, and alterations in bone microstructure and strength indices.

## MATERIALS AND METHODS

### Patient population

This study, a single-center retrospective cohort analysis, was carried out at the Sekishin-kai Kawasaki Clinic, Kanagawa, Japan. Approval for the study protocol was granted by the Showa University Ethics Committee (H25-40), ensuring adherence to the principles of the Declaration of Helsinki. Patients’ informed consent was obtained by using an opt-out approach.

At our dialysis clinic, routine DXA scans are performed on dialysis patients to assess fracture risk. The study included dialysis patients aged 18 years or older who underwent routine DXA scans at Sekishin-kai Kawasaki Clinic between September 2018 and October 2021. Exclusion criteria were: (i) patients who had already been treated with anti-osteoporosis medication; and (ii) patients who had not undergone any follow-up DXA examinations. Of the 462 dialysis outpatients receiving dialysis therapy during the above periods at Sekishin-kai Kawasaki Clinic, 94 who had received anti-osteoporosis drugs and 92 without follow-up DXA scans were excluded. Consequently, 276 patients assessed with central DXA at both baseline and follow-up were included in the study (Supplementary data, Fig. S1). The median interval (in months) from baseline to each follow-up point is presented in Supplementary data, Table S1.

### Bone densitometry and software

Hip and whole-body DXA scans were conducted with a Discovery-A scanner (Hologic Inc., Waltham, MA, USA) at Kawasaki Clinic, adhering to the manufacturer's recommendations. 3D-DXA analyses were conducted using 3D-SHAPER software (v2.11, 3D-Shaper Medical, Barcelona, Spain). Further details on 3D-DXA methods and their validation against QCT can be found elsewhere [8, 9]. The 3D-SHAPER software delivers the following measurements for the total hip region:

• Cortical compartments at the total hip region: cortical volumetric BMD (vBMD) (mg/cm^3^), cortical thickness (mm) and cortical surface BMD (sBMD) (mg/cm^3^),
• Trabecular compartments at the total hip region: trabecular vBMD (in mg/cm^3^)
• Strength indices at the neck region: cross-sectional area (CSA) (cm^2^), cross-sectional moment of inertia (CSMI) (cm^4^), section modulus (SM) (cm^3^) and buckling ratio

### Biochemical parameters

Management of CKD-MBD followed the guidelines of the Japanese Society for Dialysis Therapy [14]. Demographic and laboratory data were collected at baseline and follow-up. As regards the assay for i-PTH, electrochemiluminescence immunoassay was used, and normal values were 10–65 pg/mL.

### Statistical approach

The demographic data are presented as median values (25th–75th percentile) for continuous variables, unless otherwise noted, and as numbers (percentage) for categorical variables. Changes in parameters at each time point were determined by calculating the difference between baseline and subsequent measurements of interest. Mixed model analysis was used to assess changes in cortical and trabecular compartments and strength indices in the hip regions over a follow-up period of up to 2.5 years. Additionally, a multivariate mixed model analysis was performed to explore the relationship between time-dependent CKD-MBD markers [serum corrected calcium (cCa) levels, serum phosphate levels, serum i-PTH levels and alkaline phosphatase (ALP)] and bone microarchitecture in the total hip region and strength indices at the femoral neck, adjusting for factors such as sex, age, dialysis duration, cause of end-stage kidney disease (ESKD), usage of vitamin D receptor activator (VDRA) and calcimimetics, and CKD-MBD markers over time. A complete-case analysis approach was adopted, with missing data (primarily due to loss of follow-up) not considered in the analysis. Participant numbers at each time point are listed in Supplementary data, Table S1.

The time-fixed variables were sex, age, dialysis vintage and cause of ESKD. Time-dependent continuous variables included serum phosphate, corrected calcium, i-PTH levels and ALP. Usage of calcimimetics and VDRA were considered as categorical, time-varying covariates.

We also performed sensitivity analyses to test the robustness of our results. We treated serum i-PTH values as a categorical variable rather than a continuous variable, and the PTH low group [Japanese Society for Dialysis Therapy (JSDT): PTH < 60; Kidney Disease: Improving Clinical Outcomes (KDIGO) PTH < 55] and PTH high group (JSDT: 240 < PTH; KDIGO 585 < PTH) were used according to JSDT and KDIGO guidelines [14, 15]. i-PTH was used as a categorical time-varying covariate for the three different types of analyses used; low, reference and high.

For all tests, the level of significance was set at *P* < .05. Statistical analyses were performed using Stata 16.0 (StataCorp LLC, College Station, TX, USA) and SAS version 9.4 (SAS Institute, Inc., Cary, NC, USA).

## RESULTS

### Characteristics and clinical parameters

**Figure 1:**
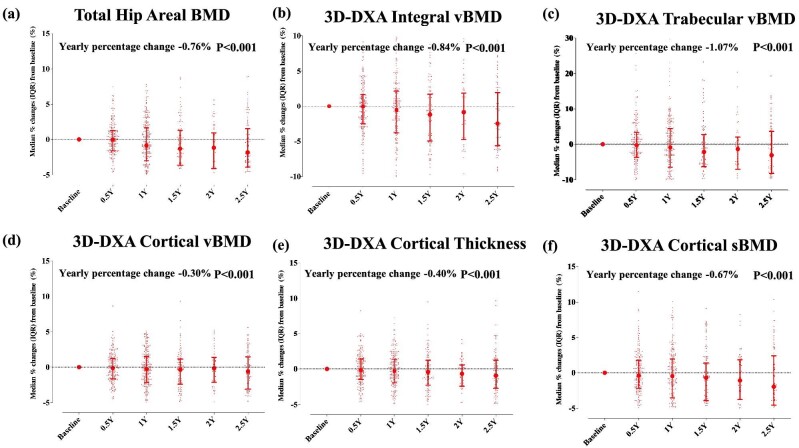
Changes at the total hip region in aBMD (**a**), integral vBMD (**b**), trabecular vBMD (**c**), cortical vBMD (**d**), cortical thickness (**e**) and cortical sBMD (**f**) in 276 dialysis patients. Data represent the median (interquartile range). *P*-value was derived from a mixed model analysis. Y, year.

**Figure 2:**
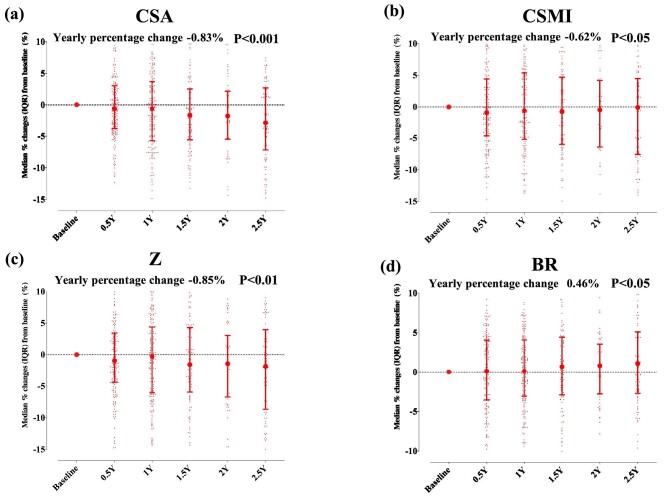
Changes at the femoral neck region in CSA (**a**), CSMI (**b**), section modulus (Z) (**c**) and buckling ratio (BR) (**d**) in 276 dialysis patients. Data represent the median (interquartile range). *P*-value was derived from a mixed model analysis. Y, year.

Table 1 showed the patients’ characteristics and clinical parameters. The median age was 61 years, and 79.3% of the patients were male. The median dialysis duration was 42 months. The most common cause of ESKD was diabetes nephropathy (44%), followed by chronic glomerulonephritis (30%). The median T-score of the whole body and femoral neck were –0.4 and –1.5, respectively. Baseline biomarkers of CKD-MBD, including serum cCa, P and i-PTH levels, were within JSDT-recommended target ranges [14]. The CKD-MBD parameters and from baseline over up to 2.5 years are shown in Supplementary data, Fig. S2. The proportion of patients within JSDT-recommended CKD-MBD target ranges is shown in Supplementary data, Fig. S3. Mixed model analysis revealed significant reductions in both cortical and trabecular compartments over the study period, alongside aBMD in the total hip region of dialysis patients (Fig. 1). Estimated bone-strength indices also showed significant deterioration, except for the buckling ratio, in the femoral neck regions (Fig. 2). The buckling ratio, an index of cortical stability, exhibited significant positive changes, suggesting decreased cortical stability in the hip regions.

**Table 1:** Baseline clinical and biochemical characteristics of 276 dialysis patients.

	Total patients (*n* = 276)
**Demography and clinical characteristics**	
Age (years)	61 (50–71)
Males, *n* (%)	219 (79)
Hemodialysis therapy, *n* (%)	254 (92)
Dialysis vintage (months)	42 (16–95)
Cause of ESKD, *n* (%)	
Chronic glomerulonephritis	83 (30)
Diabetic nephropathy	122 (44)
Hypertension	51 (18)
ADPKD	13 (5)
other	7 (3)
**BMD**	
Total whole-body BMD (g/cm^2^)	1.12 (1.05 to 1.20)
T-score (SD)	–0.4 (–1.6 to 0.7)
Z-score (SD)	1.4 (0.3 to 2.5)
Total hip BMD (g/cm^2^)	0.88 (0.79 to 0.99)
Femoral neck BMD (g/cm^2^)	0.67 (0.59 to 0.75)
T-score (SD)	–1.5 (–2.0 to –0.8)
Z-score (SD)	–0.4 (–1.1 to 0.2)
Osteoporosis^a^, *n* (%)	20 (7)
**Nutritional status**	
Body mass index	23.6 (21.1 to 27.1)
Height (cm)	165 (159 to 170)
Weight (kg)	63.8 (55.5 to 72.6)
**Circulating biomarkers**	
Albumin (g/L)	3.6 (3.4 to 3.8)
Albumin-corrected calcium (mg/dL)	8.8 (8.4 to 9.1)
Phosphate (mg/dL)	5.5 (4.8 to 6.4)
ALP (U/L)	227 (181 to 306)
i-PTH (pg/mL)	211 (142 to 345)
**Medications, *n* (%)**	
Calcimimetics	135 (49)
Etelcalcetide	26 (9)
Cinacalcet	50 (18)
Evocalcet	59 (21)
VDRA	84 (30)
Calcitriol (iv)	79 (29)
Calcitriol (oral)	15(5)

Continuous variables are presented as median (25–75 percentile). Categorical variables are presented as number (*n*)/percentage (%).

a The number of patients with a T-score at the hip of less than –2.5.

ADPKD, autosomal dominant polycystic kidney disease; iv, intravenous.

### CKD-MBD markers and changes in cortical and trabecular compartments at the total hip regions

Given the observed significant negative changes in the 3D components of the total hip regions over a follow-up period of up to 2.5 years, we explored the relationship between CKD-MBD parameters and 3D parameters in dialysis patients through multivariate mixed model analysis. When analyzing CKD-MBD parameters as time-dependent continuous variables, no significant correlations were found between serum cCa levels, phosphate levels and any 3D components of the hip regions. Conversely, the serum ALP level was significantly inversely correlated with areal BMD. Furthermore, a significant negative correlations were found between serum i-PTH levels and all measures of the cortical and trabecular compartments in the hip regions, except for cortical vBMD (Table 2).

**Table 2:** Association of CKD-MBD biomarkers and changes in 3D DXA parameters at the total hip regions from baseline over up to a maximum of 2.5 years based on multilevel mixed model analysis adjusting for sex, age, duration of dialysis, cause of ESKD, serum cCa levels, serum phosphate levels, serum i-PTH levels, serum ALP levels, vitamin D use and calcimimetics use.

		Coefficient (95% CI)	*P*-value
PTH (+1 SD increase)	aBMD	–0.004 (–0.006 to –0.001)	<.01
	Integral vBMD	–1.847 (–2.956 to –0.737)	<.01
	Trabecular vBMD	–0.012 (–0.020 to –0.004)	<.01
	Cortical vBMD	–0.013 (–0.027 to 0.001)	.069
	Cortical thickness	–0.0003 (–0.0001 to –0.000002)	<.05
	Cortical sBMD	–0.005 (–0.009 to –0.001)	<.05
Calcium (+1 SD increase)	aBMD	0.002 (–0.0005 to 0.004)	.117
	Integral vBMD	0.352 (–0.694 to 1.398)	.510
	Trabecular vBMD	0.413 (–0.501 to 1.327)	.376
	Cortical vBMD	0.292 (–1.243 to 1.826)	.709
	Cortical thickness	–0.0007 (–0.0029 to 0.0042)	.702
	Cortical sBMD	0.107 (–0.321 to 0.536)	.624
Phosphate (+1 SD increase)	aBMD	0.001 (–0.002 to 0.004)	.441
	Integral vBMD	0.651 (–0.525 to 1.827)	.278
	Trabecular vBMD	0.452 (–0.576 to 1.481)	.389
	Cortical vBMD	0.915 (–0.811 to 2.640)	.299
	Cortical thickness	–0.0001 (–0.0041 to 0.0039)	.975
	Cortical sBMD	0.179 (–0.305 to 0.663)	.468
ALP (+1 SD increase)	aBMD	–0.003 (–0.006 to 0.0002)	.069
	Integral vBMD	–0.624 (–2.010 to 0.761)	.377
	Trabecular vBMD	–0.231 (–1.436 to 0.974)	.707
	Cortical vBMD	–0.819 (–2.848 to 1.210)	.429
	Cortical thickness	0.0049 (–0.0096 to –0.0002)	<.05
	Cortical sBMD	–0.526 (–1.097 to 0.044)	.071

The time-fixed variables were sex, age, dialysis vintage, and cause of ESKD.

Time-dependent continuous variables included serum phosphate, corrected calcium, i-PTH and ALP levels.

Usage of calcimimetics and VDRA were considered as categorical, time-varying covariates.

CI, confidence interval; SD, standard deviation.

### CKD-MBD biomarkers and changes in estimated bone-strength indices at the neck regions

Similarly, we investigated the relationship between CKD-MBD parameters and estimated bone-strength indices at the femoral neck regions in dialysis patients using multivariate mixed model analysis. Although no significant associations were observed between serum cCa levels, phosphate levels and any estimated bone-strength indices, significant negative associations were found between serum i-PTH levels and CSA and SM, even after adjusting for sex, age, duration of dialysis, cause of ESKD, serum cCa levels, serum phosphate levels, serum ALP levels, vitamin D use and calcimimetics use (Table 3). The ALP also had a significant negative association with CSA.

**Table 3:** Association of CKD-MBD biomarkers and changes in estimated bone-strength indices at the femoral neck regions from baseline over up to a maximum of 2.5 years based on multivariate mixed model analysis adjusting for sex, age, duration of dialysis, cause of ESKD, serum cCa levels, serum phosphate levels, serum i-PTH levels, serum ALP levels, vitamin D use and calcimimetics use.

		Coefficient (95% CI)	*P*-value
PTH (+1 SD increase)	CSA	–0.006 (–0.011 to –0.001)	<.05
	CSMI	–0.010 (–0.021 to –0.0003)	.056
	SM	–0.00004 (–0.0001 to –0.000004)	<.05
	Buckling ratio	0.0002 (–0.0002 to 0.0007)	.341
Calcium (+1 SD increase)	CSA	0.003 (–0.001 to 0.008)	.150
	CSMI	0.003 (–0.007 to 0.013)	.508
	SM	0.003 (–0.002 to 0.007)	.213
	Buckling ratio	–0.019 (–0.068 to 0.030)	.441
Phosphates (+1 SD increase)	CSA	0.001 (–0.004 to 0.045)	.737
	CSMI	–0.002 (–0.013 to 0.009)	.692
	SM	–0.001 (–0.006 to 0.004)	.759
	Buckling ratio	–0.009 (–0.064 to 0.046)	.737
ALP (+1 SD increase)	CSA	–0.006 (–0.012 to –0.0001)	<.05
	CSMI	–0.012 (–0.025 to 0.001)	.082
	SM	–0.005 (–0.011 to 0.0004)	.072
	Buckling ratio	0.003 (–0.061 to 0.068)	.922

The time-fixed variables were sex, age, dialysis vintage, and cause of ESKD.

Time-dependent continuous variables included serum phosphate, corrected calcium, i-PTH, and ALP levels.

Usage of calcimimetics and VDRA were considered as categorical, time-varying covariates.

CI, confidence interval; SD, standard deviation.

### i-PTH levels and cortical and trabecular compartments at hip regions

Given that i-PTH levels showed a strong significant correlation with 3D components in the hip regions among CKD-MBD markers, we conducted two sensitivity analyses by categorizing i-PTH levels. Initially, using 60 pg/mL ≤ i-PTH ≤ 240 pg/mL as the reference range, as recommended by JSDT, no significant associations were observed between the lower PTH group (i-PTH ≤ 60 pg/mL) and any 3D variables in the multivariate mixed model analysis. However, the higher PTH group (240 pg/mL < i-PTH) showed a significant negative association with integral vBMD when compared with the reference. Subsequently, another multivariate mixed model analysis was performed using the KDIGO guidelines, which suggest a target range of approximately 2–9 times the upper normal limit [15]. Surprisingly, in comparison with the reference range (130 pg/mL ≤ i-PTH ≤ 585 pg/mL), the lower PTH group (i-PTH < 130 pg/mL) was significantly positively associated with both integral and trabecular vBMD. On the other hand, the higher PTH group (585 pg/mL < i-PTH) had significant associations across all cortical compartment variables in the hip regions (Table 4).

**Table 4:** Association of i-PTH and changes in 3D DXA parameters at the total hip regions from baseline over up to a maximum of 2.5 years based on multivariate mixed model analysis adjusting for sex, age, duration of dialysis, cause of ESKD, serum cCa levels, serum phosphate levels, serum i-PTH levels, serum ALP levels, vitamin D use and calcimimetics use.

	JSDT guideline				
	Low PTH group (PTH < 60)			High PTH group (240 < PTH)	
	Coefficient (95% CI)	*P*-value	60 ≤ PTH ≤ 240	Coefficient (95% CI)	*P*-value
aBMD	0.001 (–0.008 to 0.011)	.784	Ref	–0.003 (–0.008 to 0.002)	.189
Integral vBMD	2.136 (–2.314 to 6.586)	.347	Ref	–2.490 (–4.644 to –0.337)	<.05
Trabecular vBMD	2.300 (–1.579 to 6.179)	.245	Ref	–1.776 (–3.664 to 0.113)	.065
Cortical vBMD	0.309 (–6.198 to 6.817)	.926	Ref	–2.101 (–5.257 to 1.056)	.192
Cortical thickness	0.006(–0.009 to 0.021)	.409	Ref	–0.003 (–0.010 to 0.004)	.428
Cortical sBMD	0.473 (–1.351 to 2.298)	.611	Ref	–0.588 (–1.470 to 0.295)	.192
	KDIGO Guideline				
	Low PTH group (PTH < 130)			High PTH group (585 < PTH)	
	Coefficient (95% CI)	*P*-value	130 ≤ PTH ≤ 585	Coefficient (95% CI)	*P*-value
aBMD	0.005 (0.00004 to 0.011)	<.05	Ref	–0.022 (–0.037 to –0.007)	<.01
Integral vBMD	3.76 (1.373 to 6.151)	<.01	Ref	–9.493 (–16.385 to –2.602)	<.01
Trabecular vBMD	3.431 (1.335 to 5.527)	<.01	Ref	–5.298 (–11.372 to 0.776)	.087
Cortical vBMD	1.704 (–1.811 to 5.219)	.342	Ref	–12.225 (–22.383 to –2.068)	<.05
Cortical thickness	0.004 (–0.004 to 0.012)	.358	Ref	–0.034 (–0.057 to –0.011)	<.01
Cortical sBMD	0.567 (–0.411 to 1.544)	.256	Ref	–4.952 (–7.769 to –2.135)	<.01

The time-fixed variables were sex, age, dialysis vintage and cause of ESKD.

Time-dependent continuous variables included serum phosphate, corrected calcium, i-PTH and ALP levels.

Usage of calcimimetics and VDRA were considered as categorical, time-varying covariates.

CI, confidence interval.

### i-PTH levels and estimated bone-strength indices at the neck regions

Similarly, we investigated the relationship between i-PTH levels (as categorical variables) and estimated bone-strength indices at the femoral neck regions in dialysis patients. When employing JSDT criteria, neither the lower PTH group nor the higher PTH group was significantly associated with any estimated bone-strength indices. On the other hand, when using the KDIGO guidelines, the higher PTH group had significant negative associations with CSA, CSMI and SM (Table 5).

**Table 5:** Association of i-PTH and changes in changes in estimated bone-strength indices at the femoral neck regions from baseline over up to a maximum of 2.5 years based on multivariate mixed model analysis adjusting for sex, age, duration of dialysis, cause of ESKD, serum cCa levels, serum phosphate levels, serum i-PTH levels, serum ALP levels, vitamin D use and calcimimetics use.

	JSDT guideline				
	Low PTH group (PTH < 60)			High PTH group (240 < PTH)	
	Coefficient (95% CI)	*P*-value	60 ≤ PTH ≤ 240	Coefficient (95% CI)	*P*-value
CSA	0.007 (–0.012 to 0.027)	.455	Ref	0.004 (–0.005 to 0.013)	.381
CSMI	–0.008 (–0.050 to 0.034)	.716	Ref	0.010 (–0.010 to 0.031)	.332
SM	0.004 (–0.015 to 0.023)	.691	Ref	0.004 (–0.005 to 0.013)	.362
Buckling ratio	–0.101 (–0.308 to 0.107)	.341	Ref	0.016 (–0.085 to 1.118)	.750
	KDIGO guideline				
	Low PTH group (PTH <130)			High PTH group (585 < PTH)	
	Coefficient (95% CI)	*P*-value	130 ≤ PTH ≤ 585	Coefficient (95% CI)	*P*-value
CSA	0.008 (–0.002 to 0.018)	.125	Ref	–0.042 (–0.072 to –0.012)	<.01
CSMI	0.010 (–0.013 to 0.033)	.395	Ref	–0.072 (–0.139 to –0.006)	<.05
SM	0.008 (–0.002 to 0.018)	.125	Ref	–0.034 (–0.064 to –0.005)	<.05
Buckling ratio	–0.101 (–0.214 to 0.011)	.078	Ref	0.146 (–0.182 to 0.473)	.384

The time-fixed variables were sex, age, dialysis vintage and cause of ESKD.

Time-dependent continuous variables included serum phosphate, corrected calcium, i-PTH and ALP levels.

Usage of calcimimetics and VDRA were considered as categorical, time-varying covariates.

CI, confidence interval.

## DISCUSSION

In this retrospective study of 276 dialysis patients, we found a marked deterioration in both cortical and trabecular bone compartments, as well as aBMD at the hip regions. Similarly, over a maximum observation period of 2.5 years, the estimated bone-strength indices at the neck regions significantly worsened. Multilevel mixed model analysis revealed that serum cCa levels and phosphate levels had no significant association with changes in any 3D parameters or estimated bone-strength indices. On the other hand, serum i-PTH levels were significantly negatively correlated with aBMD, integral vBMD, trabecular vBMD, cortical thickness, cortical sBMD, CSA, CSMI and SM. The serum ALP also had significant negative associations with cortical thickness and CSA. In a sensitivity analysis treating i-PTH levels as time-dependent categorical variables, the high PTH group (i-PTH > 240 pg/mL) demonstrated a significant negative correlation with integral vBMD compared with the reference group (60 pg/mL ≤ i-PTH ≤ 240 pg/mL), following JSDT guidelines. When applying the KDIGO criteria, the higher PTH group (i-PTH > 585 pg/mL) had significant negative associations with areal BMD, integral vBMD, cortical vBMD, cortical thickness and cortical sBMD compared with the reference group. Notably, the lower PTH group (i-PTH < 130 pg/mL) showed a positive significant correlation with integral vBMD and trabecular vBMD.

In the human skeleton, cortical bone makes up about 80% of the total bone mass, with trabecular bone constituting the remaining 20% [16]. Cortical bone, being denser and harder than trabecular bone, primarily serves to protect soft tissues. The ratio of cortical to trabecular bone varies across different skeletal locations [17, 18]. Additionally, bone turnover rates differ among various bones and within long bones, influenced by the trabecular–cortical ratio, as well as factors including sex, age, paracrine factors and medication use [4].

Given these facts and considering that hip fractures are most common among dialysis patients as shown in the epidemiological study [19], analyzing the hip region is crucial to understanding the underlying mechanisms. Regarding cortical bone loss in dialysis patients, a previous study using QCT showed decreased total hip cortical mass and volume during a 2-year follow-up period [20]. Our findings expand this previous evidence, and we found a decrease in cortical vBMD, cortical thickness and cortical sBMD [5, 20]. Additionally, regarding longitudinal changes in trabecular bone, Malluche *et al*. showed an increase in trabecular volume, but not mass, at the total hip [20]. As BMD was bone mass per volume, this was supported by our finding showing a decrease in trabecular vBMD. To our knowledge, this study is the first to demonstrate that estimated bone-strength indices at the neck regions worsen over a maximum follow-up of 2.5 years. Bone strength, estimated by 3D-DXA finite element (FE) models incorporating detailed bone characteristics as accurately as QCT, accurately reflects femur strength and provides as a predictor of hip fracture risk [10]. Thus, the mechanism of dialysis patients having higher hip fracture risk may be explained by a marked deterioration in both cortical and trabecular bone compartments at hip regions, leading to decreased bone strength. Preserving both cortical and trabecular bone in the hip region may contribute to preventing hip fractures in dialysis patients. When starting anti-osteoporosis therapy in dialysis patients meeting osteoporosis criteria, the choice of treatments effective for both cancellous and cortical bone within the hip region, such as denosumab, would be favorable [11].

It was well-documented that better control of CKD-MBD biomarkers leads to better clinical outcomes, especially in reducing cardiovascular events, as clinical management in dialysis patients [21–23]. While cross-sectional studies have demonstrated an inverse correlation between serum PTH levels and measures such as cortical density, area, thickness and strength-strain index, longitudinal data on the relationship between time-varying PTH levels and changes in hip-bone microstructures in dialysis patients remain quite limited [24]. This study using longitudinal data found no significant associations between serum phosphate and calcium levels and changes in 3D components or estimated bone strength at the hip region. Conversely, i-PTH levels were significantly negatively associated with both cortical and trabecular bone components and estimated bone-strength indices, indicating that controlling i-PTH levels might be more crucial than managing calcium and phosphate in the bone health of dialysis patients. From another perspective, therapies that lower PTH levels to the recommended range have been linked to improved bone properties and fewer fractures in patients with secondary hyperparathyroidism [25], indicating that elevated PTH levels are detrimental to bone health in dialysis patients. On the other hand, regarding the potential harm of excessively low PTH levels, a population-based study from the JSDT Renal Data Registry found no significant difference in fracture outcomes between the parathyroidectomy (PTx) group and the cinacalcet group, despite PTx achieving significantly greater reductions in i-PTH levels compared with cinacalcet (with median i-PTH levels of 83 pg/mL in the PTx group and 218 pg/mL in the cinacalcet group) [26]. Besides, a recent study using Dialysis Outcomes and Practice Patterns Study (DOPPS) showed that low PTH had a significant association with reduced hip fracture risk [27]. Our findings, which revealed more benefits than harms to bone health in the low PTH group, lower than typically recommended thresholds according to the KDIGO criteria, might support this. However, skeletal responsiveness to PTH may vary by race [28], further study is required to verify whether our results can be extrapolated to other races.

Some limitations should be discussed when interpreting the results of the present study. First, this study, employing retrospective design, does not permit the determination of causality. Additionally, the study could not evaluate some important factors, including bone turnover markers, such as 25-Hydroxyvitamin D, Tartrate-Resistant Acid Phosphatase 5b (TRAP5b) and C-Terminal Telopeptide of Type I Collagen (CTX). Second, the generalizability of the findings is limited due to the retrospective, single-center study design. Third, despite the study population not being large, to our knowledge, our sample size and duration of follow-up surpass those of prior research in examining the relationship between time-dependent CKD-MBD biomarkers and the microstructure of hip bones in patients undergoing dialysis. Fourth, there was an absence of data from bone biopsies for the examination of histological alterations. However, it is important to note that a bone biopsy from the iliac crest may not fully reflect the hip region's condition, which is particularly critical in dialysis patients. This discrepancy arises from varying bone turnover rates, attributed to differences in cortical and trabecular content ratios across bones [4, 29, 30]. Conversely, our use of 3D-SHAPER software for direct hip region analysis provides significant insights into managing bone diseases in dialysis patients. Finally, we could not assess the associations between fracture incidents and bone microstructures.

In conclusion, this study involving 276 dialysis patients showed significant deterioration in both cortical and trabecular bone compartments, along with aBMD at the hip regions, over a maximum observation period of 2.5 years. A similar trajectory was seen in the estimated bone-strength indices at the neck regions. Multivariate mixed model analysis revealed that neither serum cCa nor phosphate levels were significantly associated with changes in 3D parameters or estimated bone-strength indices. In contrast, serum i-PTH levels were significantly inversely correlated with areal BMD, integral vBMD, trabecular vBMD, cortical thickness, cortical vBMD, CSA, CSMI and SM. Sensitivity analyses confirmed the robustness of our results and indicated that low PTH may link with favored impacts on hip bone structures. These findings suggest that elevated PTH levels contribute to the deterioration of cortical and trabecular bone, as well as reduced bone strength at the hip, potentially leading to an increased incidence of hip fractures. Effective management of PTH levels may therefore play a crucial role in hip-bone microstructure in dialysis patients, potentially mitigating bone disease.

## Supplementary Material

sfae240_Supplemental_Files

## Data Availability

The datasets used and/or analyzed during the study are available from the corresponding author on reasonable request.
