# Supplementary material for: Association between CKD-MBD and hip-bone microstructures in dialysis patients
Source: Clin Kidney J. 2024 Aug 12;17(8):sfae240. doi: 10.1093/ckj/sfae240 (PMC11345638; doi:10.1093/ckj/sfae240)
Supplement: sfae240_Supplemental_Files [file sfae240_supplemental_files.zip › 578 suppl mat.docx]

**Supplemental Table 1. The median interval (months) from baseline to each measurement.**

| **Time points (Y)** | **N** | **Median interval, month (IQR)** |
| --- | --- | --- |
| **0.5** | 186 | 6.1 (5.8 - 6.3) |
| **1** | 222 | 12.1 (11.8 - 12.5) |
| **1.5** | 111 | 18.3 (18.0 - 18.7) |
| **2** | 59 | 24.3 (24.0 - 24.6) |
| **2.5** | 98 | 30.0 (30.0 - 30.3) |

Abbreviations: Y, year; IQR, range from 25th to 75th percentile; M, month.

**Supplemental Figure 1. Patient disposition**


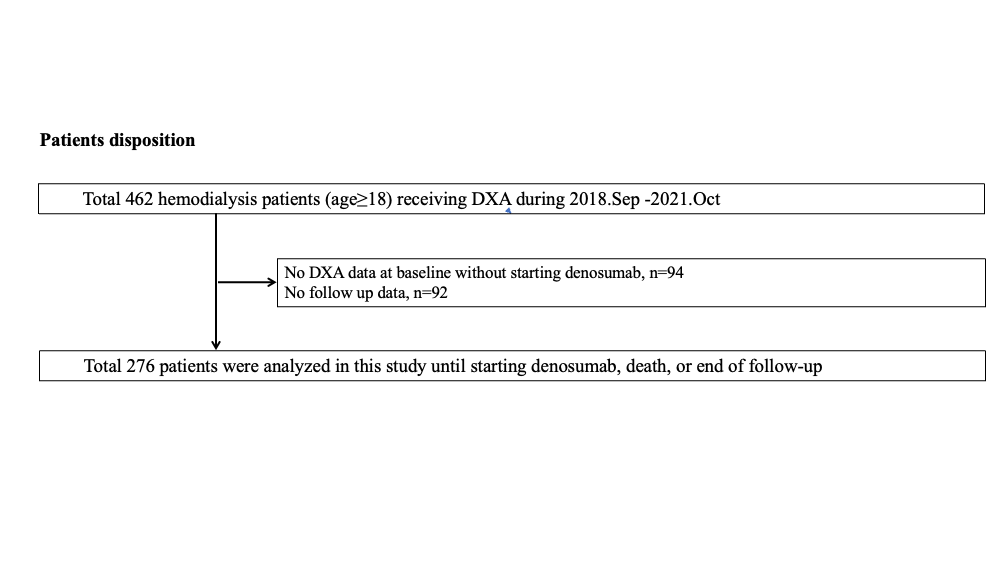


**Supplemental Figure 2.** Time courses of the CKD-MBD parameters and ALP from baseline over up to 2.5 years.


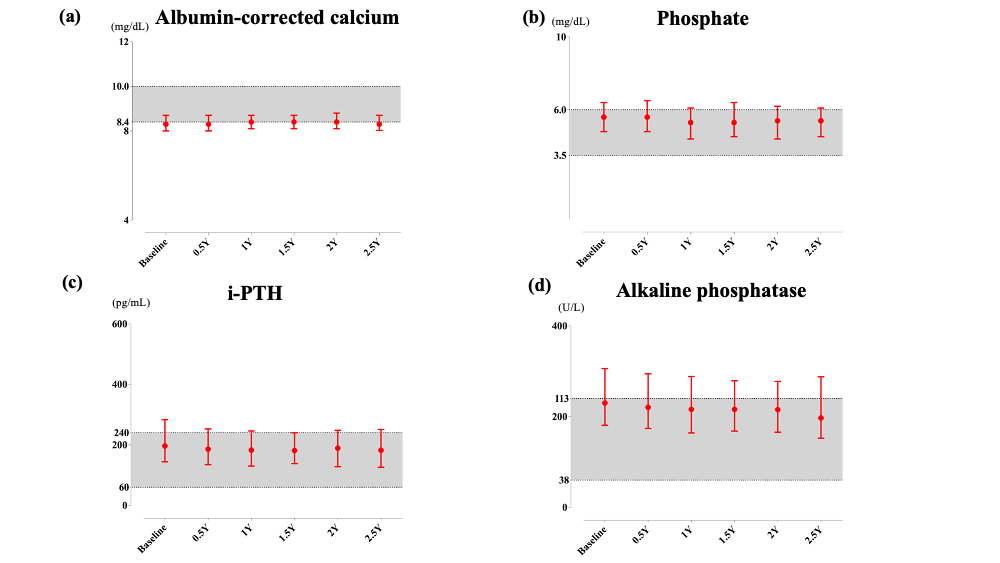


Data represent the median (IQR). The gray shaded area represents the reference value (recommended by JSDT). Abbreviation: i-PTH, intact-parathyroid hormone ALP, total alkaline phosphatase.

**Supplemental Figure 3.** Proportion of patients within JSDT-recommended CKD-MBD target ranges from baseline up to 2.5 years

**
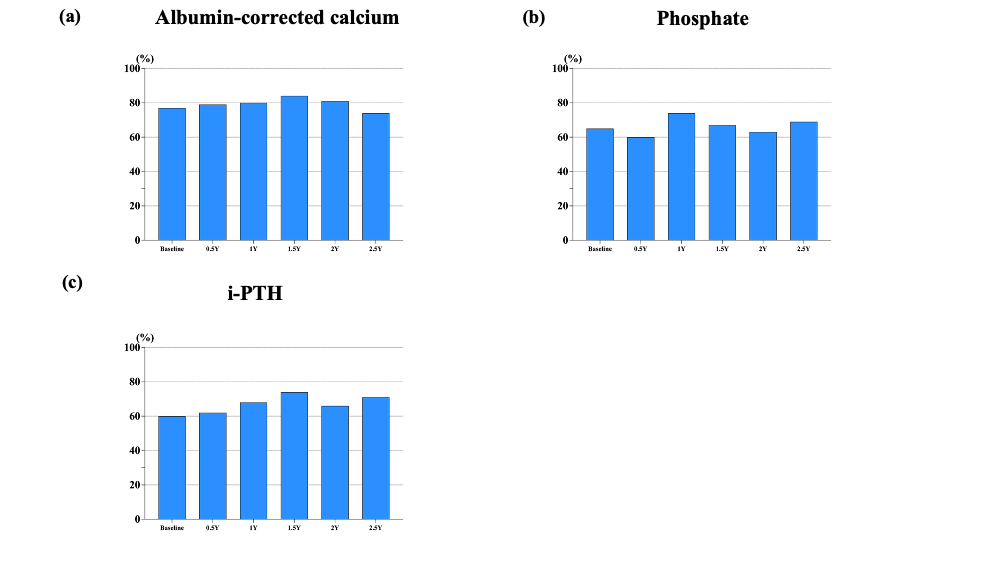
**

Abbreviation: JSDT, Japanese Society for Dialysis Therapy; i-PTH, intact-parathyroid hormone.
