# Supplementary figures and images for: Association between CKD-MBD and hip-bone microstructures in dialysis patients
Source: Clin Kidney J. 2024 Aug 12;17(8):sfae240. doi: 10.1093/ckj/sfae240 (PMC11345638; doi:10.1093/ckj/sfae240)

# Supplemental Figure1

## Patients disposition

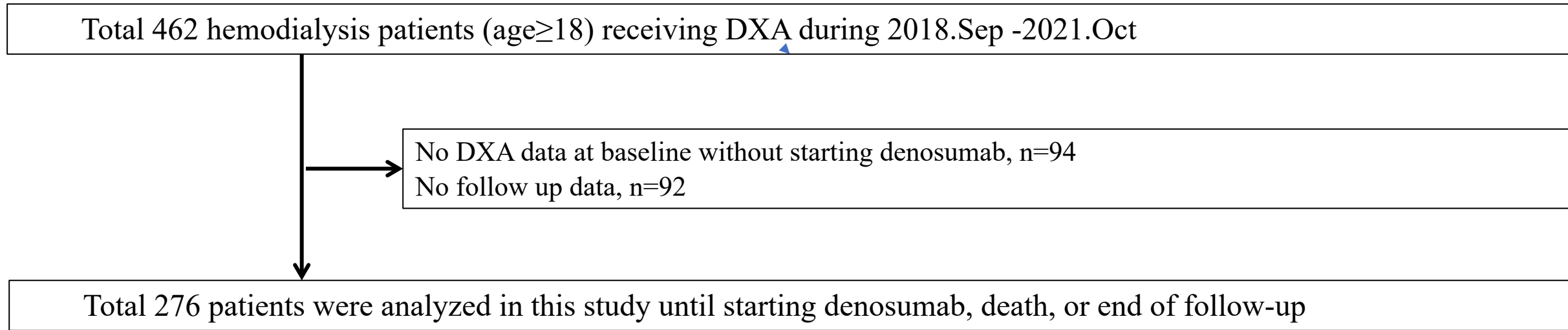

Supplement: sfae240_Supplemental_Files [file sfae240_supplemental_files.zip › Sup Fig1.pdf]

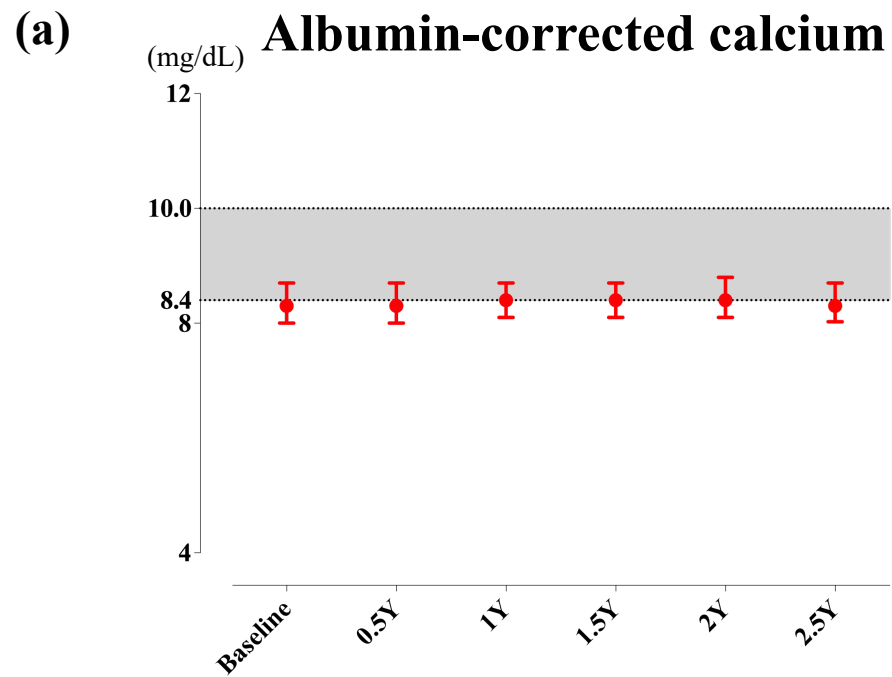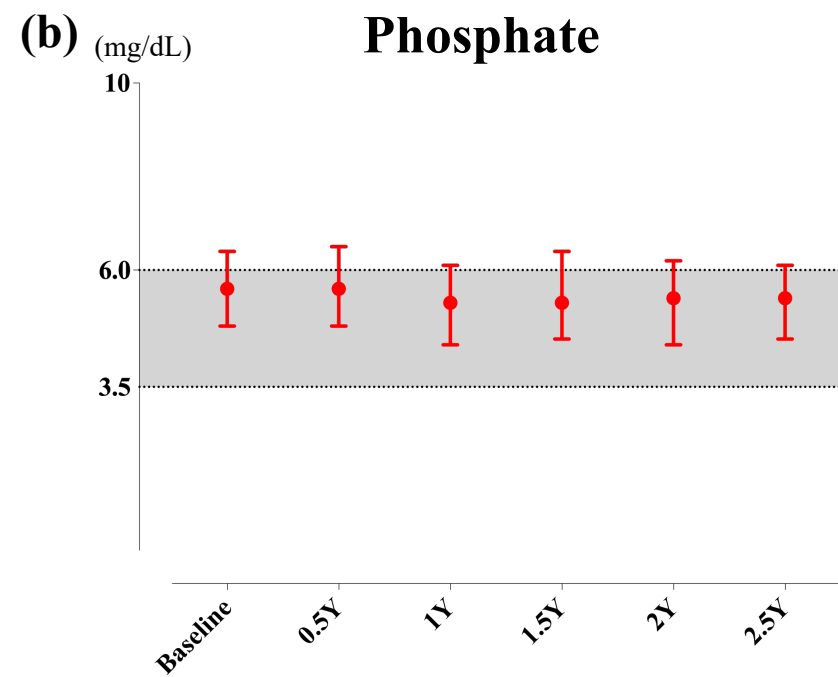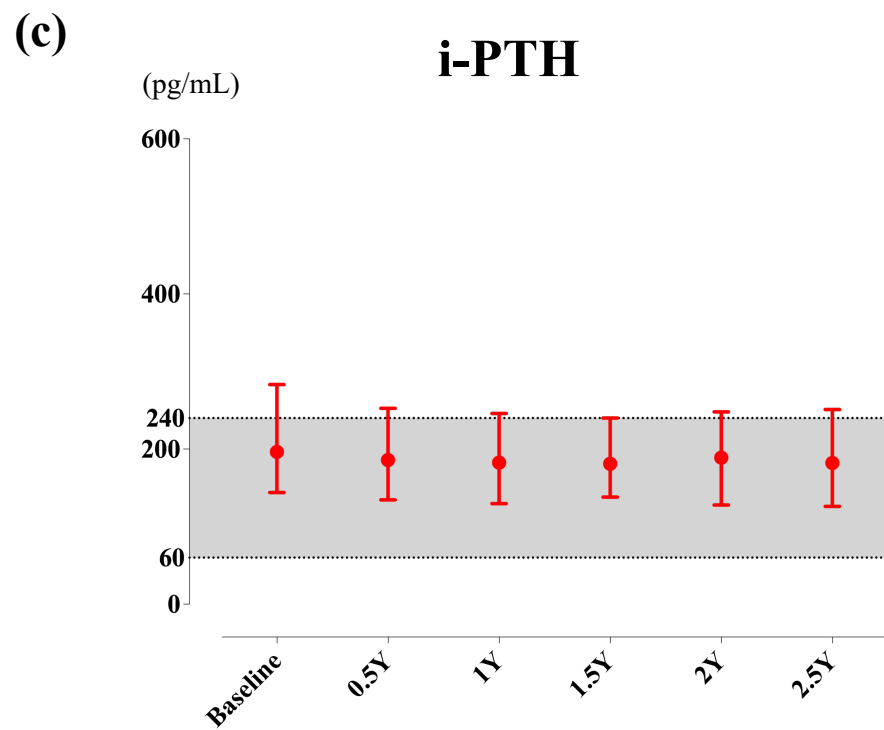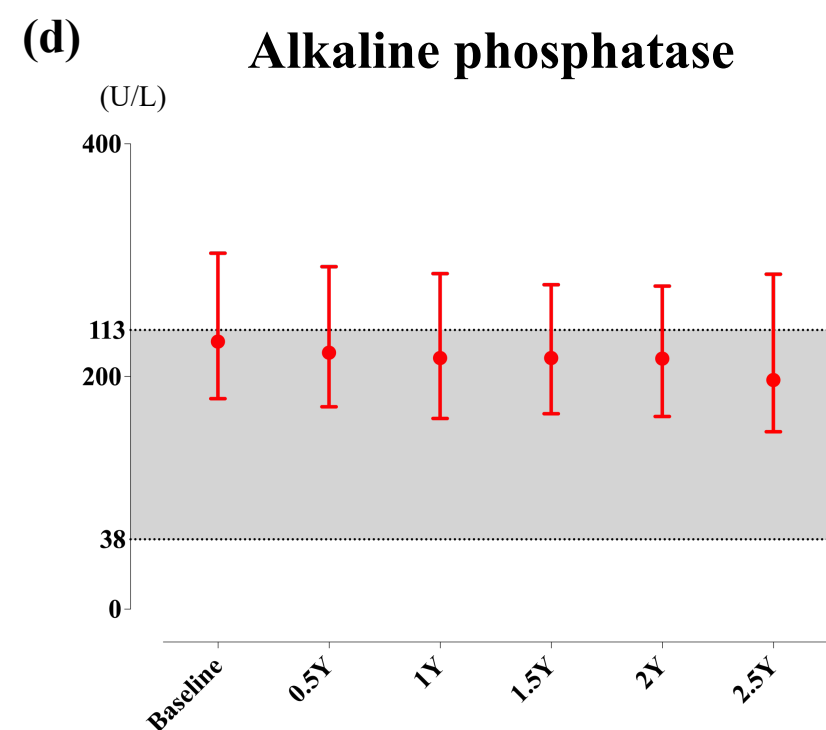

Supplement: sfae240_Supplemental_Files [file sfae240_supplemental_files.zip › Sup Fig2.pdf]

**(a) Albumin-corrected calcium**

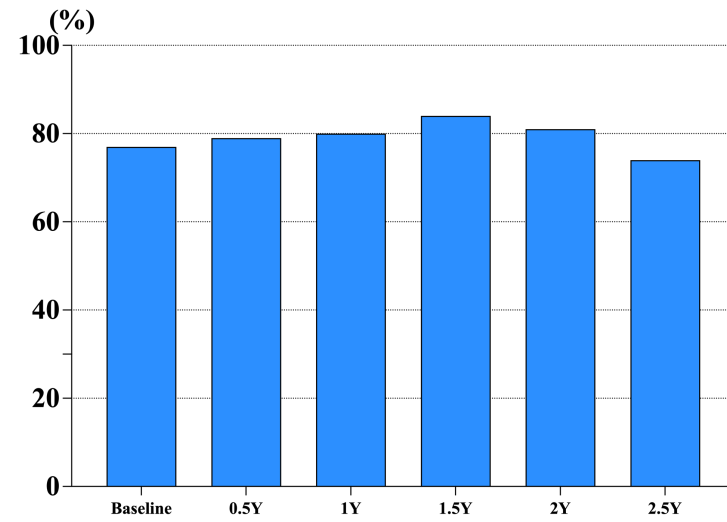

**(b) Phosphate**

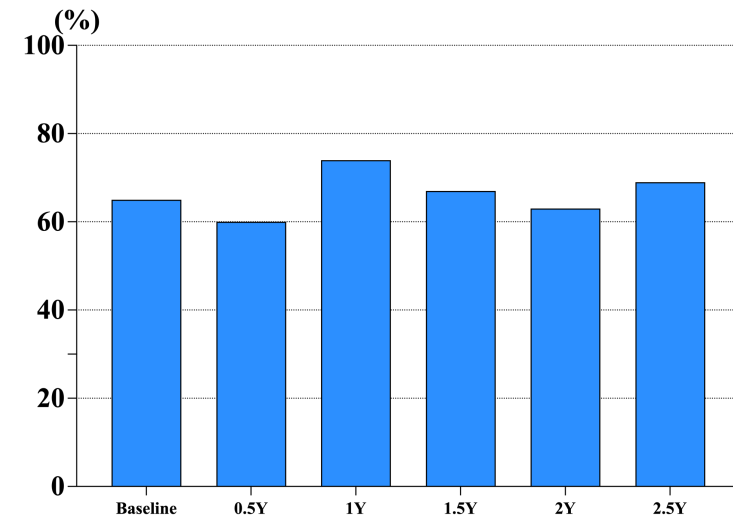

**(c) i-PTH**

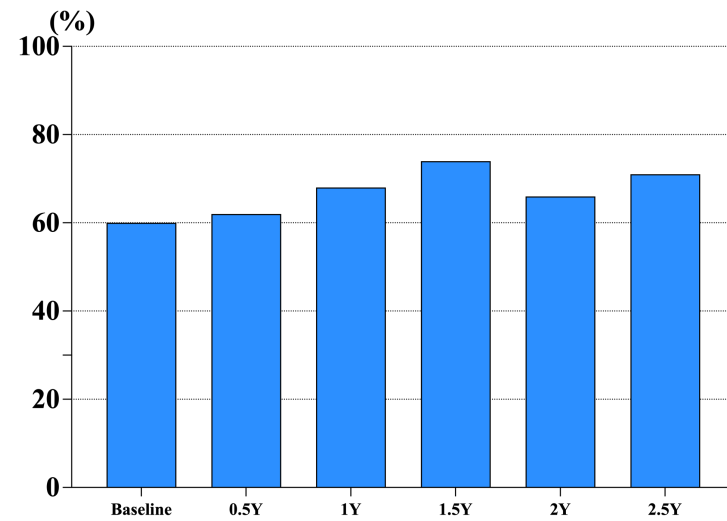

Supplement: sfae240_Supplemental_Files [file sfae240_supplemental_files.zip › Supp Fig3.pdf]
